# Change of Safe Needling Depth at Acupoint GB21 according to Posture and Breathing

**DOI:** 10.1155/2018/2308102

**Published:** 2018-01-08

**Authors:** Hongmin Chu, Jaehyun Kim, Wonbae Ha, Eunbyul Cho, Geon Kang, Seongjun Park, Jongwon Jang, Seung Bum Yang, Yeonseok Kang, Sanghun Lee, Jae-Hyo Kim

**Affiliations:** ^1^Department of Internal Medicine and Neuroscience, College of Korean Medicine, Wonkwang University, Iksan, Republic of Korea; ^2^Wonkwang University Gwangju Medical Center, Gwangju, Republic of Korea; ^3^Department of Medical History, College of Korean Medicine, Wonkwang University, Iksan, Republic of Korea; ^4^Department of Rehabilitation Medicine of Korean Medicine, College of Korean Medicine, Wonkwang University, Iksan, Republic of Korea; ^5^Department of Meridian & Acupoint, College of Korean Medicine, Wonkwang University, Iksan, Republic of Korea; ^6^Geum-il Branch Office of Wando County Health Center and Hospital, Wando, Republic of Korea; ^7^Department of Medical Non-Commissioned Officer, Wonkwang Health Science, Iksan, Republic of Korea; ^8^Research & Development, Korea Institute of Oriental Medicine, Daejeon, Republic of Korea

## Abstract

Acupoint GB21 (Jianjing) is used for treating back and shoulder pain but is associated with a risk of pneumothorax. We aimed to determine the SND (safe needling depth) at GB21 according to posture and breathing in real time. Ultrasonographic images of GB21 during normal breathing, inspiration, and expiration in a SP (sitting position) were acquired for 52 healthy volunteers. Images were also acquired during normal respiration in the PP (prone position) with arms raised and lowered. The average SND was greater for men than for women (*p* < 0.05). Analysis of variance revealed that the SND was greater for the PP than for the SP (*p* = 0.01 and *p* < 0.05, resp.). Although the SND tended to change according to posture, the average depth tended to deviate widely in some subjects. During breathing, the differences between inspiration and expiration were less than 1 mm in most subjects, but some showed differences more than 4.5 mm. The SND at GB21 was greater in overweight subjects and significantly greater in the PP and during maximal expiration. However, intragroup differences were greater than the intergroup differences. Therefore, it is dangerous to simply apply needling depth on a gender or BMI basis. The practitioner would adjust the SND by examining the individual anatomical structures.

## 1. Introduction

Acupuncture is used for treating various diseases and symptoms. If performed correctly, acupuncture is a safe medical intervention [1]. Nevertheless, acupuncture may cause side effects such as pneumothorax, nerve damage, or organ damage. Pneumothorax is the most frequently reported and most severe acupuncture-related adverse event [2–4]. Pneumothorax is known to occur when the needle hits the ribs or the upper part of the neck and back [5]. A study conducted in 2010 showed that 30% of pneumothorax resulting from acupuncture was caused by GB21 needling [2].

GB21, the acupoint of the gall bladder meridian, is intensively used in clinical procedures to treat shoulder pain, back pain, and other upper limb disorders [6]. Nevertheless, the side effects of GB21 needling have been known to exist since early times. Ancient acupuncture writings such as* Zhenjiujiayijing* [7] and* Zhenjiuzishengjing* [8] state that the needling depth at GB21 is 1.2~1.8 cm (5-6 fen: conversion of measurements according to Kim et al. [9]).* Yixuerumen *[10] states that deep insertion to GB21 causes syncope and loss of consciousness. Therefore, many researchers have tried to determine the safe needling depth or the risk depth at GB21. However, to date, studies have only been conducted using cadavers [11, 12] or CT [13] and MRI [14] analyses. Such studies are limited by factors such as cadaveric rigidity, as well as limited postural variation (i.e., supine position only) and the risk of radiation exposure during CT. Therefore, more research on GB21 needs to be conducted using ultrasonography, since it is a safe modality, which enables live imaging and thus better reflects the clinical condition.

This study examined the needling depth at GB21 and its changes according to posture and breathing by using ultrasonography, with an aim to prevent medical complications and to ensure appropriate application of acupoint GB21.

## 2. Methods

### 2.1. Participants

This study was conducted between August 1 and September 13, 2016, at the College of Korean Medicine, Wonkwang University, Iksan, Republic of Korea. Fifty-two volunteers who met the following inclusion criteria were included: (1) participants aged between 18 and 39 years and (2) agreement on participation and providing written informed consent voluntarily after receiving a clear explanation of the clinical study purposes and characteristics.

The exclusion criteria were as follows: (1) pregnant women and nursing mothers; (2) history of liver, kidney, nervous system, immune system, respiratory, endocrine, cardiovascular, or circulatory disease, tumor, or mental illness; (3) history of surgery or skin disease at the site of ultrasound imaging; and (4) abnormal shadows such as tumors found at the site of ultrasound imaging.

Agreement from the subjects to participate in the clinical study and IRB approval of Wonkwang University in Iksan, Republic of Korea (WKIRB-201607-BM-039), was obtained.

### 2.2. Location of Acupoint GB21 and Subject Posture

We palpated acupoint GB21, which is the midpoint of the line connecting the spinous process of the seventh cervical vertebra (C7) with the lateral end of the acromion, according to the WHO Standard Acupuncture Point Locations in the Western Pacific Region [15]. Thereafter, we acquired ultrasound images of the shoulder region with GB21 at the center of the probe.

The subject took a sitting or prone position on a bed while acquiring the left and right GB21 images. First, in a sitting position (StP), the subject relaxed the shoulders and breathed normally, while the GB21 images were being acquired. The subject was asked to look straight ahead, with the shoulders down, during image acquisition. Then, the subject was asked to breathe inasmuch as he/she could and to hold the breath for a while, to acquire the GB21 images at the maximal inspiratory level. The same process was used for acquiring images at the maximal expiratory level.

**Figure 1:**
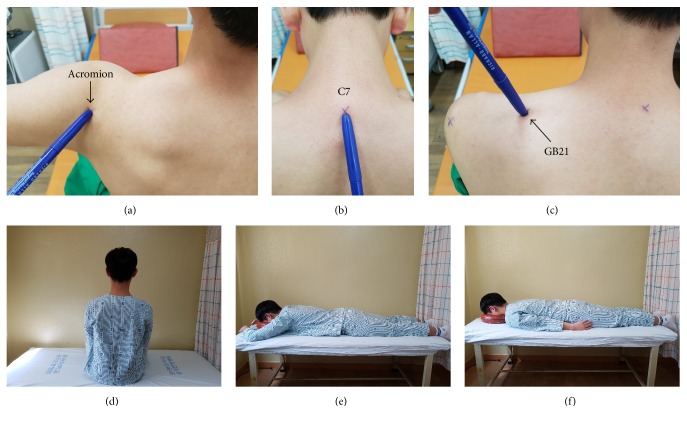
Identification of GB21 and subject posture. (a) Lateral end of the acromion. (b) Spinous process of the seventh cervical vertebra. (c) GB21, in the posterior region of the neck, at the midpoint of the line connecting the spinous process of the seventh cervical vertebra (C7) with the lateral end of the acromion. (d) Sitting position. (e) Prone position A. (f) Prone position B.

The subject then took a prone position for acquiring the left and right GB21 images. In the first prone position, defined as prone position A (PPA), the subject raised his/her arms over the head and put the hands in front of the head, with the elbows flexed at a right angle and the lateral epicondyles at the level of the ears. Thereafter, the subject was asked to place the arms parallel to the trunk, defined as prone position B (PPB), for acquiring the left and right GB21 images. Images for analyzing the change of depth at GB21 according to breathing were acquired only in the StP (Figure 1). The left and right GB21 ultrasound images were acquired using a SonoAce R7 system (Samsung Medison, Seoul, Korea) and transducers (L5-13IS-Linear Array Transducer; Samsung Medison). The subjects' weight and height were measured using an automatic height-measuring device (InBody BMS330; Biospace, Co., Seoul, Korea). The measurements were used to determine the body mass index (BMI) in kg/m^2^.

### 2.3. Analysis and Measurement

#### 2.3.1. Measurement of Safe Depths at GB21

**Figure 2:**
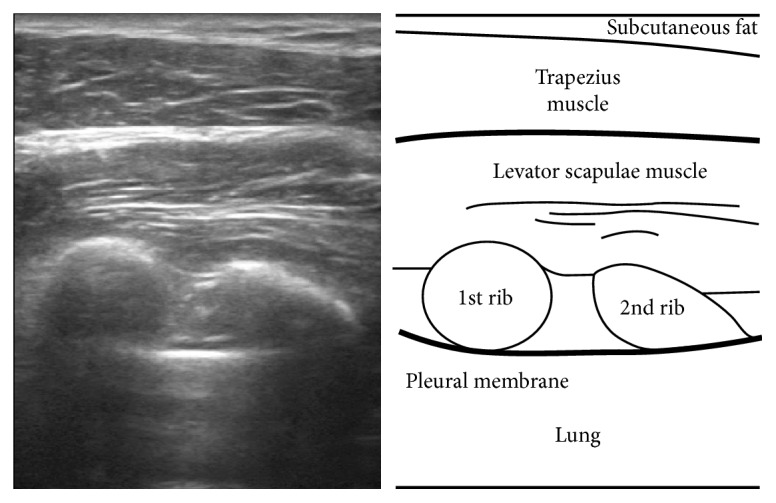
Ultrasound image of GB21. Ultrasound image, sagittal view of GB21. The trapezius muscle is seen beneath the subcutaneous fat. Underneath it is the levator scapulae muscle. The 1st and 2nd ribs lie below the levator scapulae muscle, and the pleural membrane and lung are below the ribs.

Safe needling depth at GB21 was defined as the distance between the skin and pleural membrane, which could be measured using ultrasound imaging. The probe angle was vertical to the skin over GB21 and parallel to the sagittal plane. The distance was measured using the scale mounted on the ultrasound device. Ultrasound image at GB21 is shown in Figure 2.

#### 2.3.2. Statistical Analysis

Demographic data of the subjects were presented as mean ± SD for continuous variables and percentage for categorical variables. An independent *t*-test was used to compare the depths at GB21 between the male and female groups. A paired *t*-test was used to determine the difference of depth at GB21 between maximum inspiration and maximum expiration. One-way analysis of variance (ANOVA) was used to determine the statistical differences among the safe needling depths at GB21 and different BMIs and positions. Statistical analyses were performed using IBM SPSS Statistics for Windows/Macintosh, Version 20.0 (IBM Corp., Armonk, NY, USA), and *p* < 0.05 was considered significant.

## 3. Results

### 3.1. Subject Characteristics

This study was conducted on men and women aged between 18 and 32 years. Fifty-two healthy volunteers (age range, 19–32 years; median, 22 years) participated in the study. We acquired ultrasound images of acupoint GB21 point in all subjects. The characteristics of the subjects are shown in Table 1.

### 3.2. Safe Needling Depth at GB21

Ultrasonography was used to measure the depth from the skin to the pleural membrane. We also investigated whether there was a significant difference in depth at GB21 according to sex, posture, and respiration.

#### 3.2.1. Difference in Depth at GB21 according to Sex

On average, the safe needling depth at GB21 was greater in men than in women (Table 2). The independent *t*-test between the male and female groups showed a significant difference (*p* < 0.05) even when the subject's position (StP, PPA, or PPB) changed.

#### 3.2.2. Difference in Depth at GB21 according to Posture

**Figure 3:**
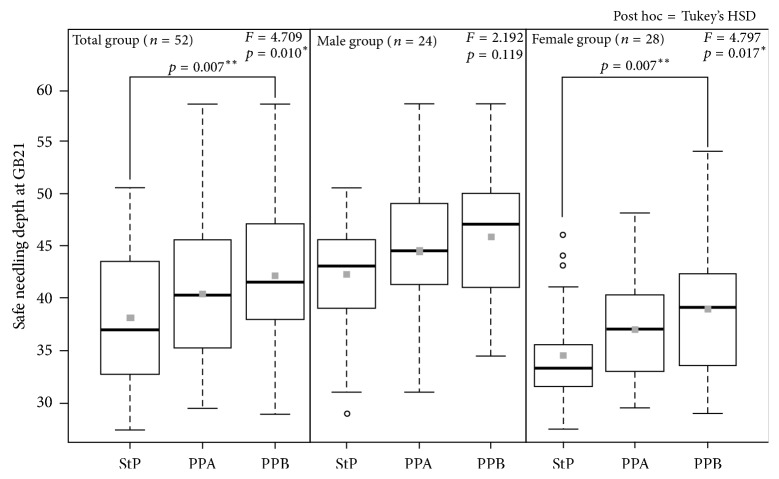
Boxplot graph of the depth at GB21. The depth at GB21 was greater in men than in women, and the depth of the pleural membrane was greater in the prone positions (PPA and PPB) than in the sitting position (StP). The gray square indicates the mean value. One-way analysis of variance was used for statistical analysis and Tukey's HSD was used for post hoc analysis. ^*∗*^*p* < 0.05, ^*∗∗*^*p* < 0.01.

Statistical analysis was performed using ANOVA to determine the difference in safe needling depth at GB21 according to subject posture. Boxplot graph of the depth at GB21 is shown in Figure 3. The safe needling depth at GB21 was significantly greater in PPB than in the StP (*p* = 0.01 and *p* < 0.05, resp.; Table 2).

A significant difference between the StP and PPB was observed in the total and female groups. No significant difference was observed between the StP and PPA, as well as PPA and PPB. No statistically significant difference according to the subject's position change was observed in the male group. However, four subjects had greater safe needling depths at GB21 in the StP than in PPA and PPB. These findings suggest a tendency that the depth at GB21 was greater in the prone positions than in the StP. However, according to our results, the deviation was large and outliers existed.

**Figure 4:**
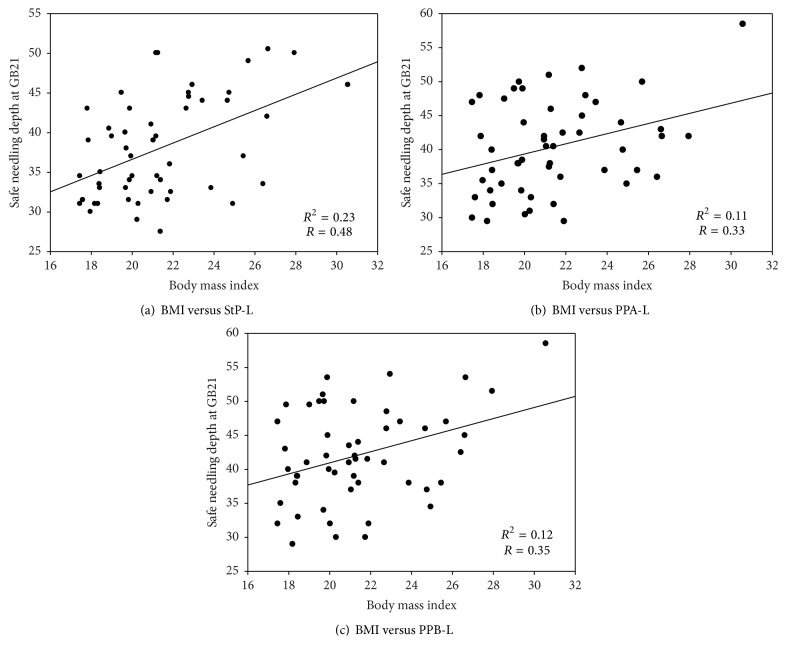
Correlation coefficient of needling depth at GB21. The depth to the pleural membrane tends to increase along with body mass index (BMI). However, the variation of depth is very wide even among subjects at the same BMI level. L: depth of the pleural membrane (lung).

We calculated the correlation coefficient between the depth of the pleural membrane. A positive correlation was observed in the StP with a higher *R*^2^ value than in PPA and PPB, as can be seen in Figure 4. However, in a subject whose BMI was 24.94 kg/m^2^, the depths were almost identical at all positions, with the thickness difference being more than 10 mm between the StP (21.5 mm) and PPA (31.5 mm). The most extreme case of depth change according to position was 19.5 mm between the StP and PPB in a subject with a BMI of 19.88 kg/m^2^. In another subject with a BMI of 21.27 kg/m^2^, the depth was greater in the StP (as much as 8.5 mm) than in PPB. Thus, although there is a statistical inclination towards a safer position, the deviation is simultaneously very high.

#### 3.2.3. Difference in Depth at GB21 according to Respiratory Changes

**Figure 5:**
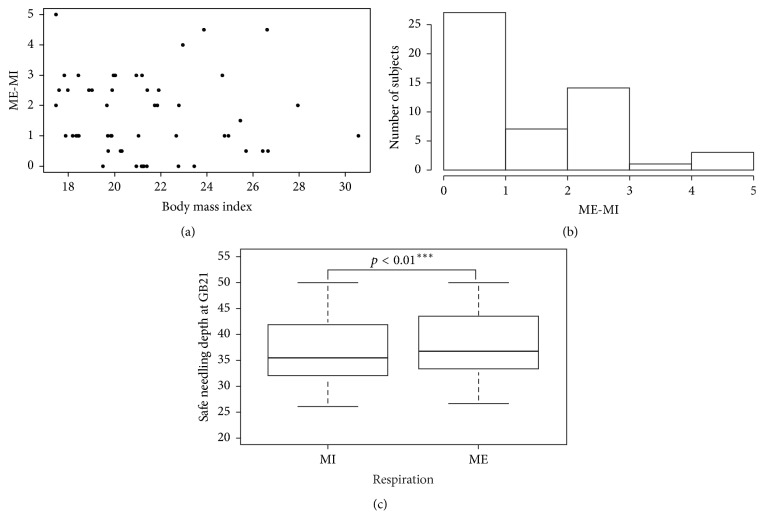
Difference between maximum expiration and inspiration. (a) Scatter plot showing the gap of depth depending on respiration and BMI. (b) Histogram showing the gap of depth at GB21 during maximum expiration and maximum inspiration. (c) Box plot graph of the depth from the skin to the pleural membrane at GB21 during maximum expiration and maximum inspiration (*p* = 1.012*e* − 06, *p* < 0.01^*∗∗∗*^). MI: maximum inspiration; ME: maximum expiration; BMI: body mass index.

In the StP, the depth of the pleural membrane at GB21 was on average lower during maximum inspiration than during maximum expiration (Table 3). Moreover, in the StP, the gap between the depth at maximum inspiration and maximum expiration was within 1 mm in most subjects. The maximal value of the difference was 5 mm in a subject whose BMI was 17.46 kg/m^2^ (Figure 5).

However, the paired-sample *t*-test showed that both groups had equal distribution (*p* = 0.9627) and met the normality criteria. The *p* value was less than 0.01, indicating a statistically significant difference. However, the difference between inspiration and expiration was less than 1 mm in most of the subjects. We should pay attention to the fact that some subjects showed a difference close to 5 mm, which was different from the average value.

#### 3.2.4. Difference in Depth at GB21 according to BMI

**Figure 6:**
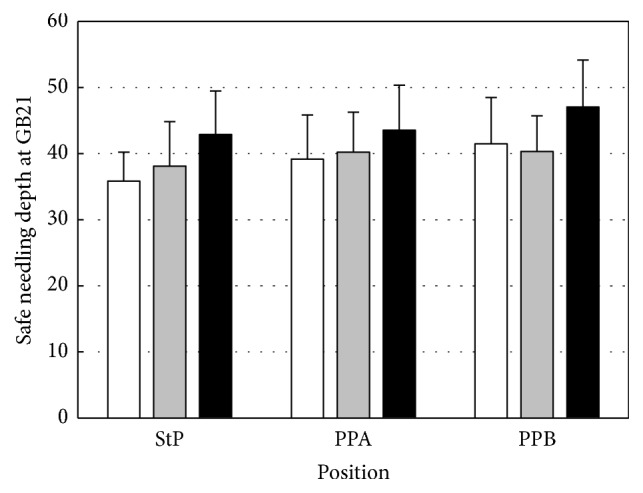
Difference of safe needling depth at GB21 according to BMI group and position. Depth of the pleural membrane according to subject position divided by BMI group. The white bar represents the underweight group; the gray bar, the normal weight group; and the black bar, the overweight group. Data are mean ± SD values of the depth of the pleural membrane. BMI: body mass index; StP: sitting position; PPA: prone position A; PPB: prone position B.

**Figure 7:**
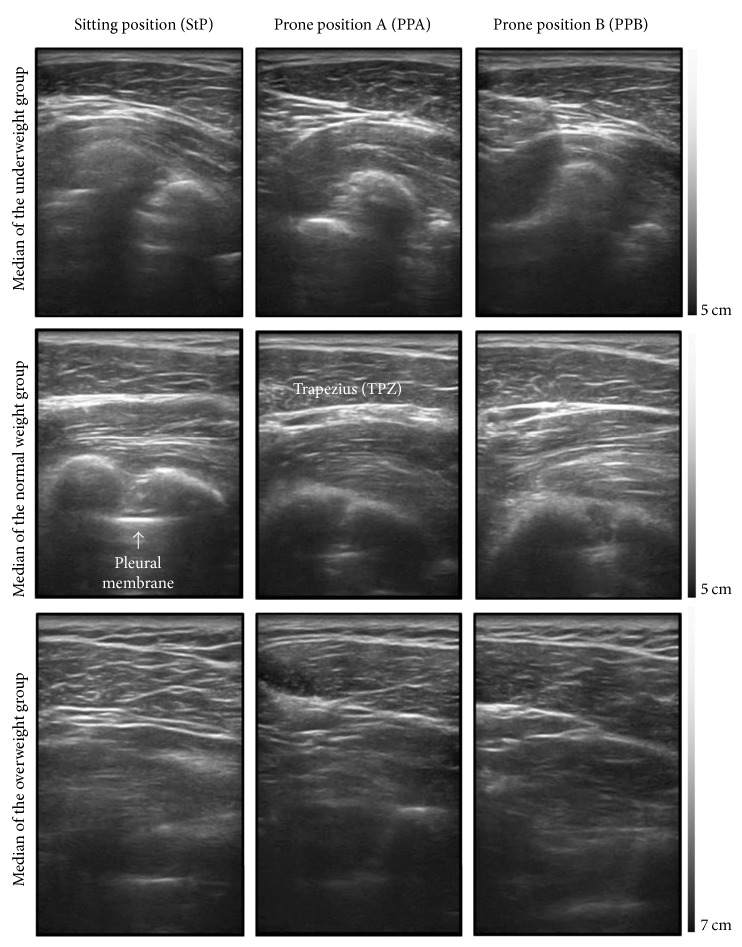
Ultrasound images of GB21 according to the body mass index (BMI) groups and positions.

The subjects were divided into three BMI groups: underweight (<18.5 kg/m^2^), normal weight (≥18.5 and <25 kg/m^2^), and overweight (≥25 kg/m^2^). The depth of the pleural membrane according to body weight tended to be greater in the overweight group than in the underweight group (Figure 6). Representative images for each weight group are given in Figure 7.

However, the tendency that “the higher the BMI is, the greater the depth is” was not seen in many subjects. The depth of the pleural membrane was lower in subjects with a BMI of 30.56 kg/m^2^ than in those with a BMI of 27.94 kg/m^2^ and 26.55 kg/m^2^. Moreover, subjects with a BMI of 26.61 kg/m^2^ (overweight group) and those with a BMI of 20.94 kg/m^2^ (normal weight group) had almost the same distance to the pleural membrane, with the difference being less than 1 mm. In PPB, the average depth in the underweight group was greater than that in the normal weight group. Therefore, although the BMI has been considered an important variable for determining the safe needling depth to date, setting BMI as a standard will be difficult.

## 4. Discussion

In this study, we used ultrasonography to measure the distance from the skin to the pleural membrane at acupoint GB21 according to posture and breathing. The average needling depth at GB21 measured by ultrasonography was 38.0 ± 6.36 mm in all the subjects, 42.27 ± 5.71 mm in men, and 34.59 ± 4.72 mm in women; the average depth in men was 7.68 ± 1.64 mm greater than that in women.

The depths according to posture were 38.08 ± 6.36 mm in the StP, 40.41 ± 6.67 mm in PPA, and 42.11 ± 6.93 mm in PPB; the average depth was 4.03 ± 0.6 mm greater in PPB than in the StP. However, the difference between the depths in the StP and PPB was close to 20 mm in an underweight subject, and the depth in the StP was 8.5 mm greater than that in PPB in a normal weight subject. Thus, there is a statistical tendency for the depth at GB21 to change according to the posture, but individual differences were significant.

The difference between the distances from the skin to the pleural membrane at GB21 according to the respiratory status was higher within a group than between the groups. That is, the interindividual difference within a group was higher than the average difference between groups, and the difference could be as high as 5 mm.

Among previous studies on the safe needling depth at GB21, four were carried out using cadavers [11, 12, 16, 17], one using MRI [14], one using CT [13], and one using ultrasonography [18]. However, only two of these studies focused solely on GB21, and these were both carried out using cadavers [12, 16]. The studies carried out using MRI or CT images were large-scale studies with more than 300 subjects, but the subjects in the study by Ma et al. [13] were aged 4–18 years. Furthermore, MRI and CT cannot consider variables such as respiratory status or posture, and particularly, studies using CT could unnecessarily expose subjects to radiation. Studies using cadavers are limited by the fact that real-time measurement of conditions such as heartbeat and vasomotion of the subjects is not possible. Ultrasonography is considered a more suitable modality for studying the safe needling depth, because it provides imaging information before and after acupuncture treatment in real time and is safe to the human body. However, Lee's study [18] using ultrasound was performed with just five men and one woman, and this small sample size and unbalanced sex ratio were its weakness. The needling depths at GB21 measured in previous studies are shown in Table 4.

Compared to our study, the previous studies conducted using cadavers or CT imaging measured the needling depths only in the supine position; therefore, the depths at GB21 were deeper in those studies than in our study. Moreover, compared to the depth measured by ultrasonography in our study, the assumed safe needling depth in the study using cadavers [16] showed that the danger of pneumothorax caused by piercing the pleural membrane was 53% and 63% in male and female, respectively.

On account of the limitations of the previous studies, we have increased the number of subjects and investigated the change in needling depth with posture and breathing, which have not been considered in previous studies. We changed the subject's posture three times and measured the significant changes in anatomical structures according to maximal inspiration and expiration in the StP. This is significant in that it reflects the actual condition of the subjects receiving acupuncture treatment, unlike in the previous studies.

As a result, we found that there is a difference in the safe needling depth according to posture and respiration as well as sex and BMI. We found that the depth studied with conventional safe needling depth can also have risk of pneumothorax.

The results of this study suggest that the location of the pleural membrane of healthy male subject in twenties (a 99% confidence interval) is located in the subcutaneous 39.27 mm~45.27 mm region, and in healthy female subjects in twenties, the position of the pleura membrane is presumed to be located at the 32.35 mm~36.60 mm site (a 99% confidence interval). This means that the there is a probability of damaging the pleural membrane when injecting over 4.0 mm, which is outside the confidence interval. It has lower value than previous studies

However, this study was conducted on healthy twenties, so further study is needed to see whether the results of this study can be applied to patients and other age groups. Also, in the analysis of ultrasonographic images in our research, seven out of 13 subjects who were excluded because of uncertain imaging were included in the overweight group with BMI of 25 or more, including the subject with the highest BMI (31.64 kg/m^2^). If ultrasonography had better resolution or specification, we could have obtained more images in the overweight group.

In addition, we confirmed the previously unknown correlation of the depth of pleural membrane with posture and breathing but did not confirm the parameters to predict safe needling depth. This is presumably because the safe needling depth of GB21 is determined not only by sex and posture, but also by various factors such as the degree of individual muscle development and body shape characteristics.

Although the safe needling depth at GB21 is measured, the standard deviation is greater than 6 mm. It indicates that it is hard to measure the needling depth by using only anatomical palpation. This suggests that high-risk acupoints may require the assistance of an imaging device. Not only acupoint GB21 but also other high-risk acupoints may induce organ damage. Therefore, further research should be undertaken to examine the risk of organ damage by analyzing other high-risk acupoints. There is a need to develop a practical determination method for the safe needling depth to prevent adverse events.

## 5. Conclusion

This study aimed to determine the safe depth of acupoint GB21. We aimed to develop a safety guideline for determining a consistent and safe needling depth, which could be used according to subject posture and respiration. The needling depth changed significantly with a change in posture. A difference in depth was also observed between maximal inspiration and expiration, but it was not clinically significant. From the result of this study, the safe needling depth of GB21 in twenties is less than 35 mm in male and 30 mm in female. Compared to the depth measured by ultrasonography in this study, the assumed safe needling depth in the previous study using cadavers showed that the danger of pneumothorax caused by piercing the pleural membrane was 53% and 63% in male and female, respectively. However, we showed that the needling depths can easily deviate from the mean and standard deviation in many subjects. Therefore, it is dangerous to simply apply needling depth on a gender or BMI basis. In the future, additional research on safe needling depth through image data of subjects at various ages is needed, and the practitioners are recommended to consider not only BMI, gender, and posture but also the individual anatomical structure characteristics together when deciding needling depth.

## Figures and Tables

**Table 1:** Characteristics of the study population.

	Men	Women	Total
Sample	24	28	52
BMI^*∗*^ (kg/m^2^)	22.27 ± 3.36	20.66 ± 2.27	21.4 ± 2.95
Underweight (<18.5 kg/m^2^)	2	9	11
Normal weight (≥18.5 & <25 kg/m^2^)	17	17	34
Over weight (≥25 kg/m)^2^	5	2	7

^*∗*^BMI: body mass index.

**Table 2:** Depth at GB21 according to subject positions and sex.

	Pleural membrane (Lung)			
	Total	Male	Female	*p* value^(1)^
	(*n* = 52)	(*n* = 24)	(*n* = 28)	(male versus female)
PPB	42.11 ± 7.00	45.85 ± 5.75	38.89 ± 6.20	0.000^*∗∗∗*^
PPA	40.41 ± 6.74	44.46 ± 6.08	36.95 ± 5.00	0.000^*∗∗∗*^
StP	38.08 ± 6.42	42.27 ± 5.71	34.48 ± 4.36	0.000^*∗∗∗*^
*F-*value^(2)^	4.079^*∗*^	2.192	4.797^*∗*^	—

*StP: sitting position; PPA: prone position A; PPB: prone position B*; data are represented as the mean ± SD (mm); (1) statistical significance was tested using *t*-tests between male and female subjects. ^*∗*^*p* < 0.05,  ^*∗∗∗*^*p* < 0.001; (2) statistical significance was tested using one-way analysis of variance among the position groups.

**Table 3:** Depth at GB21 according to respiratory changes in the sitting position.

	Pleural membrane	
	MI	ME
Total (52)	36.85 ± 6.44	38.13 ± 6.40
Male (24)	41.20 ± 5.78	41.92 ± 5.92
Female (28)	33.10 ± 4.25	34.89 ± 4.81

MI: maximum inspiration; ME: maximum expiration.

**Table 4:** A literature survey of the depth at GB21.

Author(s) and year	Sample size	Measuring tool	Parameter	Result of GB21 depth (mean ± SD)
	(male/female)			
*Zhenjiujiayijing*. 256	—	—	—	12.0 mm (5 *fen*^*∗*^)
*Yixuerumen.* 1220	—	—	—	18.0 mm (6 *fen*^*∗*^)

Zhang et al. 2002	57 (24/33)	Cadaver	Sex	(Dangerous needling depth) 55.96 mm
				(Safe needling depth) 39.17 mm
Cui et al. 2004	20 (17/3)	Cadaver	Sex	(Lung) 52.3 ± 9.4 mm
				(Trapezius) 20.2 ± 9.4 mm
Chen et al. 2006	46 (30/16)	Cadaver		(Dangerous needling depth) 62 mm
				(Safe needling depth) 41 mm
Chou et al. 2011	394 (198/196)	MRI	Sex, age, BMI, BW, height	(Underweight) 37 mm, (ideal BW) 51 mm
				(Overweight) 61 mm, (obesity) 70 mm
Lee 2015	6 (5/1)	Ultrasound	Sex, BMI, BW	(Lung) 20~34 mm
Ma et al. 2016	319 (205/114)	CT	Sex, age, BMI, BW	(Boy) 28.01 ± 9.37 mm
				(Girl) 26.61 ± 8.81 mm

Our study	52 (24/28)	Ultrasound	Gender, BMI, SP, RES	(Pleural membrane)
				StP 38.08 ± 6.36 mm
				PPA 40.41 ± 6.36 mm
				PPB 42.11 ± 6.93 mm

^**∗**^ *fen*: traditional Chinese unit of measurement. In the case of length, 1 fen is considered equivalent to 2.4 mm in Wei nation (**魏**) AD 200, and 3.11 mm in Ming nation (明) AD 1500. *BMI: body mass index; BW: body weight; SP: subject's position; RES: respiration.*
